# London Samaritan Society's Convalescent Homes

**Published:** 1891-07-25

**Authors:** 


### London Samaritan Society's Convalescent Homes.

We have recently paid a visit to the convalescent homes established at Sandgate by the London Samaritan Society (the offices of which are at 98, High Street, Homerton, E.), the occasion being the dedication of the new buildings now being erected to take the place of the dozen houses in which the patients are at present quartered. Starting as a very small affair, in fact, in the sending down some fifteen years ago of one or two poor old men and women to the seaside by Mr. J. J. Jones, the Managing Director and Treasurer, the work so rapidly increased that a concentration of forces became necessary, and Sandgate, with its many advantages as a health resort, was settled on as the headquarters for the homes. Although some 180 beds are provided in the houses now occupied, this has been found wholly insufficient, and Mr. Jones, with his characteristic go and energy, has gone straight to work and set about building a new home at Beach Rocks capable of accommodating 230 patients, into which, when finished, all the patients from the various houses dotted about Sandgate will be removed. At the present time one-half of this annex has been completed and the other half is in progress. Funds, however, are greatly needed to complete the buildings properly. We were greatly pleased at the homely air which characterised the whole place, and although certain rules necessarily have to be carried out strictly, there is yet a total absence of cast-iron routine which so often has a depressing effect on patients. In addition to this, the homes are entirely unsectarian, and all "sorts and conditions of men" and women are admitted. It will, therefore, be seen that the work appeals to all persons who are philanthropically inclined, and those who cannot afford to give money can help by sending clothing for the patients, many of whom arrive at the homes with nothing but what they stand up in—that often consisting of naught but outer garments.

